# Mechanical Properties and Structures of Clay-Polyelectrolyte Blend Hydrogels

**DOI:** 10.3390/gels4030071

**Published:** 2018-08-30

**Authors:** Hiroyuki Takeno, Shiori Nagai

**Affiliations:** 1 Division of Molecular Science, Graduate School of Science and Technology, Gunma University, 1-5-1 Tenjin-cho, Kiryu, Gunma 376-8515, Japan; t13301113@gunma-u.ac.jp; 2 Gunma University Center for Food Science and Wellness, 4-2 Aramaki, Maebashi, Gunma 371-8510, Japan

**Keywords:** clay, blend hydrogels, toughness, polyelectrolyte, synchrotron small-angle X-ray scattering

## Abstract

Our recent studies have shown that the hydrogels prepared by blending clay, a dispersant of clay, and a polyelectrolyte (sodium polyacrylate (PAAS)) possess excellent mechanical properties. In order to clarify the mechanism of the toughness, we have so far investigated the effects of the composition, molecular mass of the polymer, and kinds of polymers on the mechanical properties. This study has focused upon the mechanical properties and structures of the clay/PAAS gels using three kinds of smectite clay minerals such as synthetic hectorite (laponite XLG), saponite (sumecton-SA), montmorillonite (kunipia-F), whose particle size becomes larger according to the sequence. Laponite/PAAS and sumecton/PAAS gels were quite tough for high compression, whereas kunipia-F/PAAS did not gelate. In comparison between sumecton/PAAS gel and laponite/PAAS gel, the mechanical property of the former gel was poorer than that of the latter gel due to the inhomogeneous distribution of clay platelets in the gel. Synchrotron small-angle X-ray scattering experiments revealed that their clay platelets laid down in the stretching direction under elongation. Furthermore, it was found that sumecton/PAAS gel under elongation was arranged with an interparticle distance of ~6.3 nm in the direction perpendicular to the stretching. Such local ordering under elongation may originate in local aggregation of sumecton platelets in the original state without elongation.

## 1. Introduction

Hydrogels composed of clay and polymer have attracted widespread interest due to their potential applications in various fields. Incorporation of the clay into polymer hydrogel results in improvement of the mechanical properties [1,2,3]. Most of such clay–polymer nanocomposite hydrogels with excellent mechanical performance have been prepared by the in situ polymerization method [4,5,6,7,8]. Their studies have shown that the tensile stress increased with the increase of clay concentration [9,10] and interactions between clay and polymer are attributed to noncovalent bonds, e.g., hydrogen bonds between the amide groups and silanol groups (Si–OH) and/or siloxane units (Si-O-Si) play an important role on the cross-link of clay-poly(alkyl acrylamide) nanocomposite hydrogel [11]. In addition, the effect of clay type (synthetic hectorite, fluorinated hectorite, natural montmorillonite, or sepiolite magnesium silicate) on the mechanical performance of the nanocomposite hydrogels has been examined, so that it has been shown that synthetic hectorite (laponite XLG)—the polymer nanocomposite hydrogel has the best mechanical performance [11].

Recently we have succeeded in fabricating mechanically tough hydrogels composed of clay, a dispersant of clay, and an ultrahigh molecular mass polyelectrolyte such as sodium polyacrylate (PAAS), which have been prepared by blending them [12]. The clay/PAAS hydrogels are tough and thus suitable for high compression, and have a very large swelling ratio. So far we investigated effects of molecular mass of polymers and the composition on the mechanical properties of the hydrogels. As a consequence, it has been clarified that the factors such as dispersion of clay platelets, use of ultrahigh molecular mass polymers higher than a few million, and favorable interactions between clay and polymers are important for accomplishment of the toughness [13,14,15]. 

In this study, we investigated the mechanical properties and structures of the clay/PAAS blend hydrogels using three kinds of layered silicate minerals belonging to smectite group, such as synthetic hectorite, synthetic saponite, and montmorillonite. Smectite clay minerals have a 2:1 layer structure, in which an octahedral sheet is sandwiched between two tetrahedral sheets. Although layers of these clay minerals have nearly the same thickness of ~1 nm, the dimensions are very different. As mentioned above, although the effect of the clay type on the mechanical properties of the clay–polymer nanocomposite hydrogels synthesized by the in situ polymerization method has been investigated, no studies have made for the nanocomposite hydrogels prepared by blending (blend hydrogels). In this study, our peculiar attention is to investigate the effect of clay size on the mechanical properties of the clay/PAAS blend hydrogels. 

## 2. Results and Discussion

**Figure 1:**
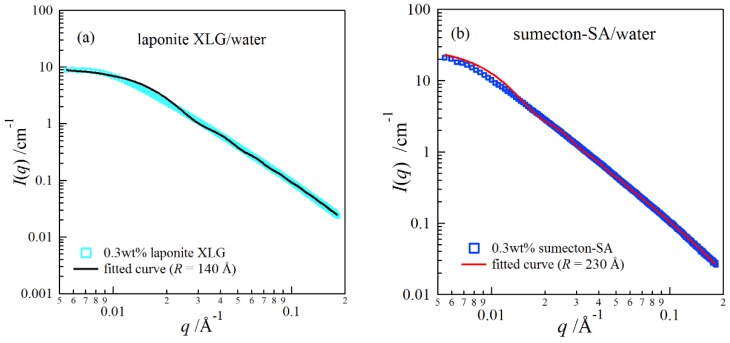
Small-angle X-ray scattering profiles for a 0.3 wt % laponite aqueous dispersion (**a**) and for a 0.3 wt % sumecton aqueous dispersion (**b**).

Firstly, we estimated the size of the clay platelets with synchrotron small-angle X-ray scattering (SAXS) for dilute clay aqueous dispersions. Assuming that the interference effects between clay platelets are negligible for a very dilute clay dispersion, the scattering intensity *I*(*q*) is expressed by

(1) $$\;I(q)\;=n_{\mathrm{clay}}V_{\mathrm{clay}}^{2}\Delta \rho^{2}P\left(q\right)$$

where *n*_clay_ and ∆*ρ* are the number density of a clay platelet with a volume of *V*_clay_ and the scattering contrast factor, respectively. Here *P*(*q*) represents the form factor of randomly distributed cylindrical particles with the radius *R* and thickness 2*H* [16,17]

(2) $$P(q)=4\int_{0}^{\pi /2\;}\left[\frac{\sin^{2}\left(qH\cos\beta \right)}{{\left(qH\cos\beta \right)}^{2}}\right]\left[\frac{J_{1}^{2}\left(qR\sin\beta \right)}{{\left(qR\sin\beta \right)}^{2}}\right]\sin\beta d\beta$$

where *J*_1_ is the Bessel function of the first order, and *β* represents the angle between *q* and the axis of the disk. Figure 1 depicts SAXS curves for dilute clay aqueous solutions, (a) a 0.3 wt % laponite solution and (b) a 0.3 wt % sumecton solution. The curves fitted with Equation (2) are in good agreement with the scattering data. The fitting analysis showed that the size of sumecton (*R* = 23 nm) was larger than that of laponite (*R* = 14 nm). 

**Figure 2:**
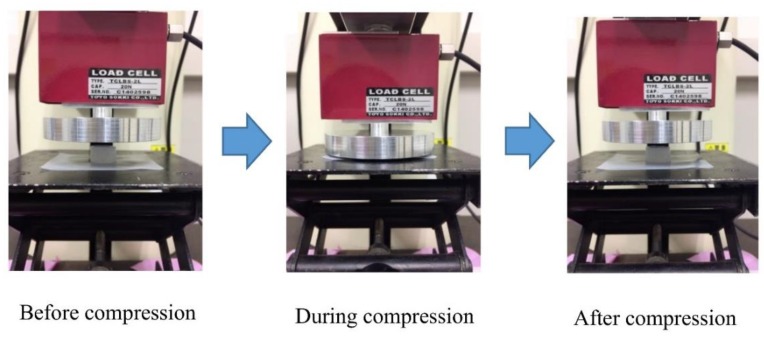
Pictures of 10 wt % sumecton/PAAS blend hydrogel in compression measurements before, during, and after uniaxial compression.

**Figure 3:**
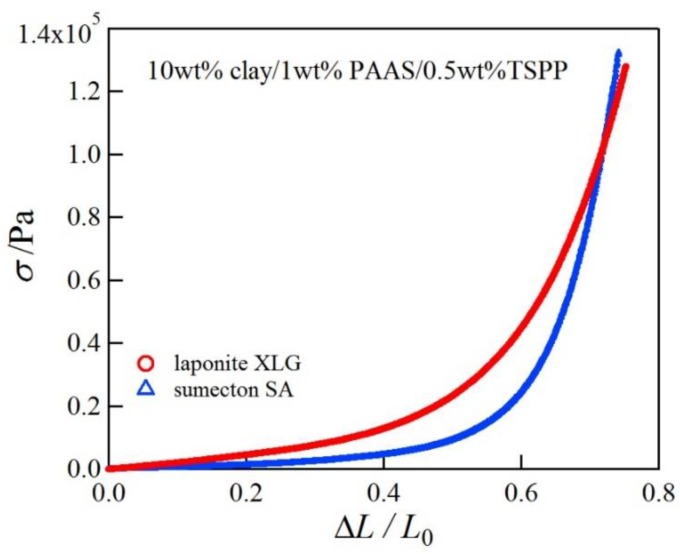
Representative compressive stress–strain curves for 10 wt % laponite/PAAS and 10 wt % sumecton/PAAS blend hydrogels.

**Figure 4:**
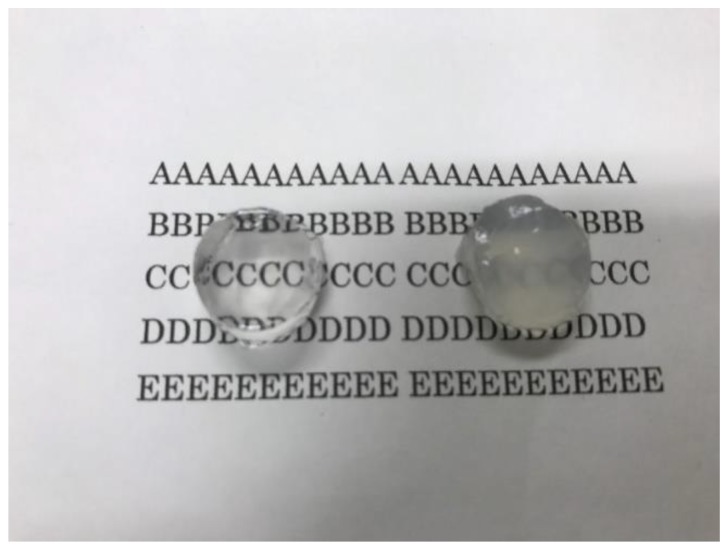
Pictures of a 5 wt % laponite/PAAS blend hydrogel (left) and a 5 wt % sumecton/PAAS blend hydrogel (right).

**Figure 5:**
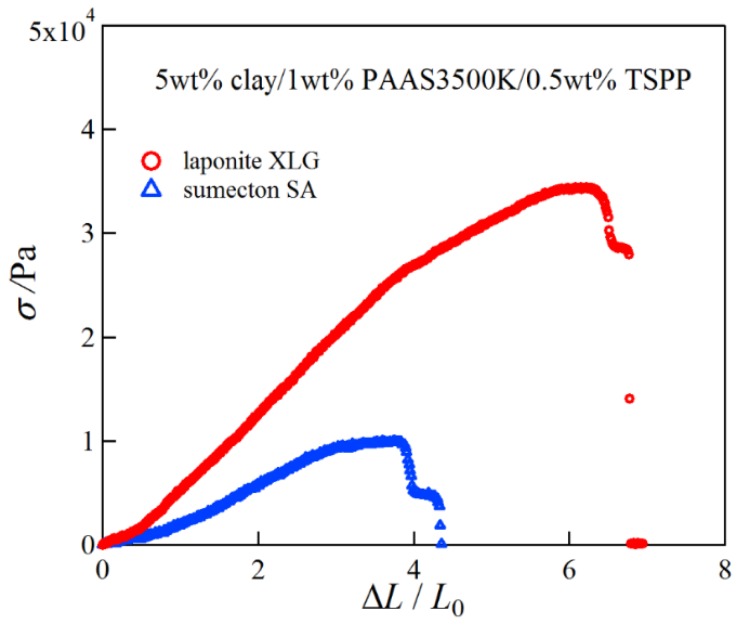
Representative tensile stress–strain curves for 5 wt % laponite/PAAS and 5 wt % sumecton/PAAS blend hydrogels.

Next we investigated the mechanical properties of the clay/PAAS blend hydrogels. Figure 2 shows pictures of a 10 wt % sumecton/PAAS gel before, during, and after compression. The sumecton/PAAS gel almost recovered its initial shape after load removal, as shown in the pictures. The behavior is the same as the laponite/PAAS gel, whose pictures were shown in the previous study [12]. The elastic behavior of the sumecton/PAAS and laponite/PAAS gels suggests incorporation of clay platelets into the polymer matrix, i.e., formation of clay/polymer nanocomposite hydrogel, where clay platelets act as a multiple cross-linker. In practice, as the gels show an alkaline nature (pH ≈ 9.9), as shown in Table 1, PAAS exists as carboxylate ions in the gel, which is expected to adsorb upon the positively charged edge of clay platelets. The comparison of compressive properties of the sumecton/PAAS and laponite/PAAS gels showed that the compressive stress of the former gel was lower than that of the latter gel (Figure 3). On the other hand, the mixture of PAAS and kunipia F with larger particle size (300~500 nm) [18] did not gelate. This result is different from that of the kunipia F-poly(*N*,*N*-dimethyl acrylamide) (PDMAA) gel prepared by the in situ polymerization method. Although the mechanical performance for the kunipia F-PDMAA nanocomposite hydrogel was lower than that of the laponite/PDMAA nanocomposite hydrogel, the former gel also showed high elongation [11]. Such a difference may come from the different preparation processes of the synthesized gel and the blend gel, i.e., as discussed in the previous paper [14], in the case of the synthesized gel, even if monomers before polymerization are added in the kunipia F dispersion, the resulting solution may not become inhomogeneous. Though the progress of polymerization may induce phase separation, or an inhomogeneous structure, the resulting gelation may give rise to freezing of the structure and prevent the inhomogeneous structure from forming. Contrary to this, in the case of the blend hydrogel, the mixture of kunipia F with large size and ultrahigh molecular mass PAAS has a tendency to become thermodynamically immiscible, because the combinatorial entropy of mixing is very small. Consequently, the effect of the clay size on the mechanical performance becomes more sensitive for the blend hydrogel. Thus, as the clay size is larger, the mechanical properties became poorer. The elastic moduli for laponite/PAAS and sumecton/PAAS gels are summarized in Table 1. The modulus of the sumecton/PAAS gel was slightly lower than that of the laponite/PAAS gel at 5 wt % clay concentration, whereas the former was much lower than the latter at 10 wt % clay concentration. The earlier studies have shown that an increase in clay concentration gives rise to a considerable increase in the elastic modulus of the gel, unless dispersion of clay platelets in the gel is significantly affected [1,12,14]. Therefore, the elastic modulus of the laponite/PAAS gel remarkably increases with the increase of the clay concentration. This behavior is attributed to the increase in the functionality of the gel that represents the number of the cross-linking points per clay platelet [12,14]. On the other hand, in the case of the sumecton/PAAS gel, the increase in clay concentration gave rise to only a slight increase in the elastic modulus. The result was attributed to poor dispersion of sumecton platelets at the higher clay concentration, as suggested from the results of transmittance in Table 1, which tends to reduce the mechanical performance. On the other hand, dispersion of sumecton platelets at the lower clay concentration may be moderately poor. As a matter of fact, the 5 wt % laponite/PAAS gel was transparent, whereas the 5 wt % sumecton/PAAS gel was translucent, as shown in Figure 4. Next, we examined the tensile properties of laponite/PAAS and sumecton/PAAS gels. Figure 5 depicts representative tensile stress–strain curves for the 5 wt % laponite/PAAS and 5 wt % sumecton/PAAS gels. The tensile strength and extension ratio for the laponite/PAAS gel was better than those of the sumecton/PAAS gel. 

**Figure 6:**
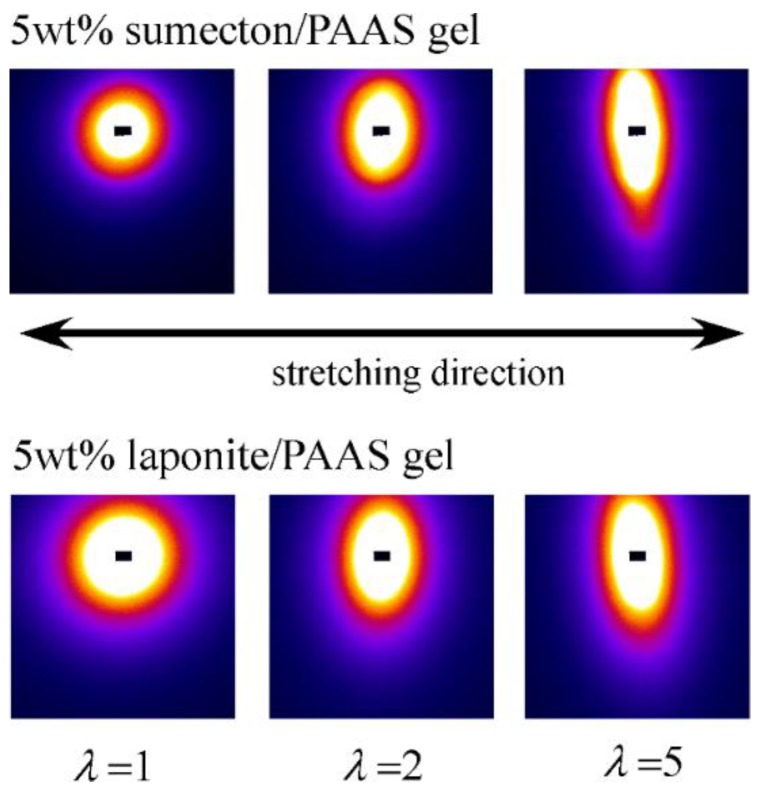
SAXS images for 5 wt % sumecton/PAAS blend hydrogels (upper) and 5 wt % laponite/PAAS blend hydrogels (lower).

**Figure 7:**
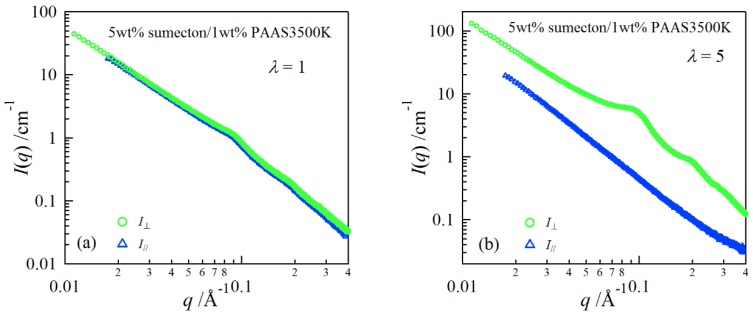
SAXS profiles in the directions parallel and perpendicular to the stretching for a 5 wt % sumecton/PAAS gel at the elongation ratio of 1 (**a**) and 5 (**b**).

**Figure 8:**
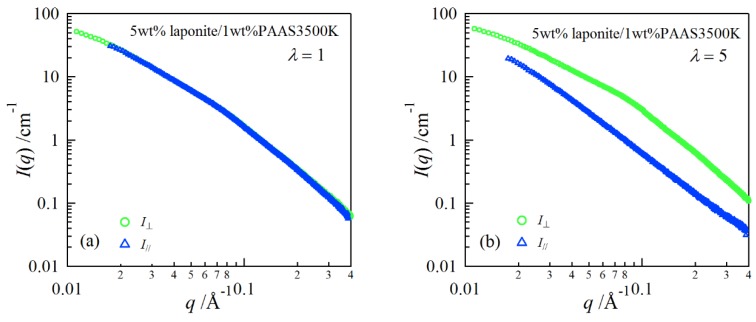
SAXS profiles in the directions parallel and perpendicular to the stretching for a 5 wt % laponite/PAAS gel at the elongation ratio of 1 (**a**) and 5 (**b**).

**Figure 9:**
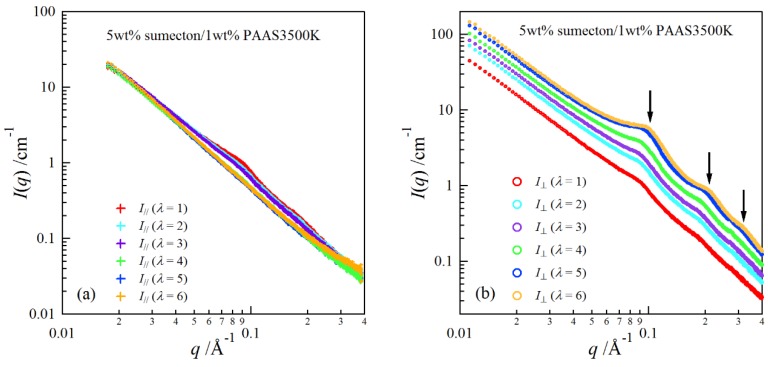
SAXS profiles at different elongation ratios in the parallel direction (**a**) and in the perpendicular direction (**b**) for the sumecton/PAAS gel.

In order to investigate structure of the gels during stretching, synchrotron SAXS experiments were performed. Although the clay/PAAS blend hydrogel is a four-component system and therefore the scattering intensity of the gel is given by the sum of the partial scattering functions between the respective components, in fact the partial scattering function for the clay–clay components is dominant in the X-ray scattering, as mentioned in the previous paper [14]. Namely, we can clearly see the structure of the clay platelets from the SAXS intensity of the gel. This is because the clay platelets are composed of heavier atoms, which have larger X-ray scattering length [17]. Figure 6 depicts two-dimensional patterns for laponite/PAAS and sumecton/PAAS gels during elongation. The SAXS patterns for both gels are isotropic before stretching (see the SAXS pattern at the extension ratio *λ* = 1), reflecting that the structure is isotropic before stretching, whereas they show elliptic patterns with a longer axis in the vertical direction during stretching. The anisotropic character increased with the increase of the stretching ratio. The elliptic patterns suggest that the clay platelets lie down towards the stretching direction under elongation, considering an inverse relationship between the object size and the scattering angle in scattering theory [19]. As a matter of fact, the scattering intensity for both gels in the perpendicular direction was stronger than that in the parallel direction, as shown in Figure 7 and Figure 8. These results also support the above interpretation. The scattering curves of the sumecton/PAAS gel in the perpendicular direction at high elongation had multiple small peaks or shoulders (Figure 9). The ratio of the peak position was 1:2:3 and the first peak was observed at *q* ≈ 0.1 Å^−1^. Thus, sumecton/PAAS gel may be arranged in the perpendicular direction with an interparticle distance of ~6.3 nm at high elongations. Such regular structure may reflect local aggregation of clay platelets in the original state (undeformed state), which contributes to lower the mechanical performance of the gel. 

## 3. Conclusions

In this study, we examined the mechanical properties and structures of hydrogels composed of sumetite clay minerals and PAAS. The dimensions of the clay platelets significantly affects the mechanical performance of the gels, i.e., as the dimensions of the clay platelets becomes larger, the mechanical performances of the gels are lowered. The mechanical strength of sumecton/PAAS gel was slightly worse than that of laponite/PAAS gel. The lowering of the mechanical strength for the sumecton/PAAS hydrogel was found to come from the structural inhomogeneity in the hydrogel. The SAXS experiments under elongation revealed that the clay platelets for both laponite/PAAS and sumecton/PAAS gels laid down in the stretching direction. The latter gel showed a regular structure with an interparticle distance of ~6.3 nm in the direction perpendicular to the stretching, which may be originated from the local aggregation in the original state. 

## 4. Experiments

### 4.1. Sample and Sample Preparation

In this study, we used three kinds of laponite XLG (RockWood Ltd., Newry, UK), sumecton-SA, and Kunipia-F (Kunimine Industries Co., Ltd., Tokyo, Japan) as clay samples, a dispersant of clay platelets (tetrasodium pyrophosphate (TSPP)) and sodium polyacrylates (PAAS) with a weight-averaged molecular mass of 3.5 × 10^6^ from Wako Pure Chemical Industries, Ltd., Osaka, Japan. The hydrogel was prepared in the same manner as described in the previous study [13,14], except for using a sonicator (QSONICA Q55, Waken Tech co., Ltd., Kyoto, Japan) for the dispersion of clay platelets. The final concentration of PAAS and TSPP in the hydrogels was 1 wt % and 0.5 wt %, respectively. 

### 4.2. Transmittance and pH Measurements

Transmittance measurements were performed for laponite and sumecton gels with a thickness of 6 mm using a He–Ne laser with a wavelength of 632.8 nm. pH values of the gel surface were measured using a pH meter (Horiba, LAQUA F-71, Kyoto, Japan) with a pH electrode (Horiba, ISFET 0040-10D).

### 4.3. Compression and Tensile Measurements

Compression tests were conducted for the gels with a cylindrical shape of a diameter of 14 mm and a thickness of 8 mm using AIKOH Engineering 1305NR (Osaka, Japan) with a load cell of 20 N and FA1015B Force Analyzer Explorer III at a compression speed of 10 mm/min. Tensile measurements were performed with an ORIENTEC TENSILE TESTER STM-20 (Tokyo, Japan) at a compression speed of 10 mm/min. Stress was calculated using a cross-sectional area of an undeformed gel. The elastic modulus *E* was obtained from the slope of the stress–strain curves at the small strains, and the average value of *E* was evaluated from three tests. 

### 4.4. Synchrotron Small-Angle X-ray Scattering (SAXS)

Synchrotron small-angle X-ray scattering (SAXS) was performed at beam line 6A and 10C of the photon factory (PF) of the High Energy Accelerator Research Organization (KEK) in Japan. The SAXS experiments were conducted with a wavelength of *λ* = 1.5 Å, and the samples for the measurements were put in an aluminum spacer with a thickness of 1 mm using a very thin Kapton film as a window. The SAXS data was collected by a two dimensional detector (PILATUS-2M or PILATUS-1M) and then circularly averaged to obtain the scattering curves as a function of *q* defined as follows,

*q* = 4*π*sin(*𝜃*/2)/*λ* (3)

where *𝜃* denotes the scattering angle. The scattered intensity obtained thus was corrected for the background scattering, and then reduced to the absolute unit using a glassy carbon [20]. 

## Figures and Tables

**Table 1:** Characteristic of *x* wt % clay/PAAS hydrogel.

Clay	pH	*E*/× 10^3^ Pa	Transmittance/%
Sumecton (*x* = 5)	9.95	2.4 ± 0.2	9.96
Laponite (*x* = 5)	9.90	3.7 ± 0.1	84.7
Sumecton (*x* = 10)	9.92	4.7 ± 0.3	1.10
Laponite (*x* = 10)	9.86	26 ± 7.3	60.7
